# A Qualitative Model Demonstrating the Adaptation of Amphibians to Semi-Arid and Arid Habitats: Comparing the Green Toad *Bufotes sitibundus* (Pallas, 1771) and *Pelophylax bedriagae* (Camerano, 1882)

**DOI:** 10.3390/ani14233351

**Published:** 2024-11-21

**Authors:** Gad Degani

**Affiliations:** 1 MIGAL–Galilee Research Institute, P.O. Box 831, Kiryat Shmona 1101602, Israel; gad@migal.org.il; 2 Faculty of Science and Technology, Tel-Hai Academic College, Qiryat Shemona 1220800, Israel

**Keywords:** *Bufotes sitibundus*, *Pelophylax bedriagae*, adaptations, habitats, Mediterranean, desert environments

## Abstract

In this brief article, the green toad (*Bufotes sitibundus*) and the Levant water frog (*Pelophylax bedriagae*) were compared to better understand the adaptations needed by amphibians, specifically the green toad, to survive in arid regions and to inhabit a wide range of habitats. The information mainly comes from data gathered in Israel, a nation experiencing a shift from Mediterranean to desert ecosystems where both amphibian species can be found. Using these data, a qualitative model is put forward that showcases the differences between these two amphibians and illustrates how the green toad has adapted to arid environments. For instance, green toads travel to breeding and spawning sites during the rainy season. During this journey, they often have to cross roads, which puts them at risk of being hit by vehicles. The main distinction between the green toad and the water frog is that the green toad moves from land environments to water, while the water frog stays in its aquatic habitat for its entire life cycle.

## Simple Summary

This article compares the green toad (*Bufotes sitibundus*) and the Levant water frog (*Pelophylax bedriagae*) to explore how amphibians adapt to arid environments. The study focuses on data collected in Israel, where Mediterranean and desert habitats intersect. A qualitative model is proposed to illustrate the differences between the two species, emphasizing the green toad’s adaptations for survival in dry areas.

## 1. Introduction

**Figure 1:**
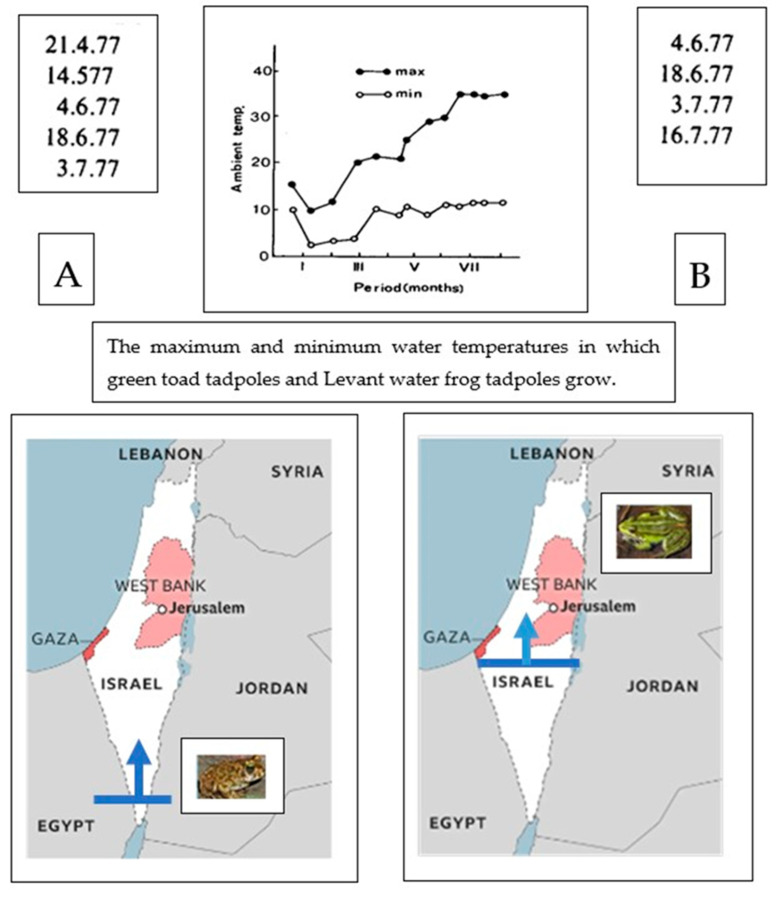
Distribution and habitat of the green toad (**A**) and the Levant water frog (**B**) in Israel, the Gaza Strip, and the Palestinian territories. The data on the green toad and the Levant water frog were collected in areas across Israel, including both natural habitats and modified environments such as agricultural fields, ponds, and urban regions. These regions provided a diverse range of ecological conditions essential for understanding the species’ distribution, behavior, and adaptability. The maximum and minimum water temperatures in which green toad tadpoles and Levant water frog tadpoles grow were measured in a winter pond (Sasa pond) where six amphibian species native to Israel are found. The environmental conditions for these species have not been studied in Israel.

The green toad (*Bufotes sitibundus*) and the Levant water frog (*Pelophylax bedriagae*) both inhabit Israel’s Mediterranean region, sometimes sharing the same habitat but different niches, and sometimes in different distribution areas (Figure 1) [1].

The geographic distribution of *Bufotes sitibundus*, commonly known as the Eastern Green Toad, has a wide geographic distribution primarily across parts of Europe and Asia. According to data from the Global Biodiversity Information Facility (GBIF), this species is predominantly found in Eastern Europe, in countries like Ukraine, Belarus, and parts of the Balkan Peninsula); Western Asia, where populations are present in regions of Turkey and around the Caspian Sea; and Northern Africa, where although less common, occurrences have been documented in northern African countries. *Pelophylax bedriagae* primarily inhabits areas in Eastern Europe and parts of Western Asia. The geographic distribution based on data from the Global Biodiversity Information Facility includes the Caucasus and surrounding regions, where the species is predominantly found in Georgia, Armenia, and Azerbaijan, which are part of its core range; Northern Iran, where occurrences have been recorded in the northern regions of Iran; and Turkey, where the frog is also present in the eastern parts of Turkey. Another study by Korzikov and Aleksanov (2018) focused on the Upper Oka Basin in Central Russia. They identified specific factors that drive the presence of amphibians in local water bodies, highlighting how environmental conditions can impact species distribution [1,2].

Comparing the distributions, habitats, ecological niches, life cycles, and physiologies of these two species in Israel can help elucidate the traits that enable amphibians to adapt to terrestrial conditions compared to less extreme climates. In addition, it can shed light on how these two species divide ecological niches amongst themselves. Israel is particularly suitable for this study because the northern part of the country features a Mediterranean climate that transitions to a semi-arid region in the center and to a desert region in the south. The green toad penetrates desert areas in Israel, where the water frog is not found [1,2].

## 2. Green Toad and Water Frog Classification

The green toad, scientifically known as *Bufotes sitibundus*, has been referred to by various names (synonyms) in different studies across its wide distribution range. Initially, the genus *Bufo* was changed to the genus *Pseudepidalea*, but in 2010, it was shown that *Pseudepidalea* is a junior synonym of *Bufotes*. *Bufo viridis*, Laurenti, 1768 [2] was later changed to *Bufotes sitibundus*. This toad species is found across various regions of Europe, Asia, and North Africa. The green toad inhabits a wide range of environments, including semi-deserts, steppes, savannas, grasslands, and various types of woodlands. This species is highly adaptable and can also be found in urban areas, agricultural landscapes, and gardens [3]. The Levant water frog, *Pelophylax bedriagae*, belongs to the family Ranidae, which is a large family of true frogs. The species is native to the Levant region, including Israel, Lebanon, Syria, and parts of Turkey. The species was originally described as *Rana bedriagae* by the German zoologist George Albert Boulenger in 1896 [4,5]. It was later reclassified under the genus *Pelophylax* due to molecular and morphological studies that differentiated this genus from other true frogs in the *Rana* genus.

The article by Dufresnes and Litvinchuk (2022) [6] examines the diversity and distribution of frogs and toads in the Eastern Palaearctic region, focusing on molecular species delimitation. The study integrates genetic data to identify and categorize various species, revealing patterns of biodiversity and potential evolutionary relationships. The findings contribute to our understanding of amphibian diversity in this region, highlighting conservation needs and the impact of environmental changes on these species. In a related study, Dufresnes et al. (2019) [7] explore the evolutionary history of Bufotes toads, reassessing their classification and genetic variation. The research emphasizes the complexity of species delineation within this group and provides insights into their evolutionary adaptations and ecological roles. The findings enhance our knowledge of amphibian diversity in this area, underscoring the importance of conservation efforts and the effects of environmental shifts on these species. In a similar study, Dufresne et al. (2019) [7] examine the evolutionary background of Bufotes toads, re-evaluating their classification and genetic diversity. This research highlights the intricate nature of species identification within this group and offers valuable perspectives on their evolutionary adaptations and ecological significance. The Bufotes species complex demonstrates a complicated taxonomic composition, with notable potential for adaptation and invasiveness [6,7]. Similarly, the Pelophylax species complex presents a complex taxonomic structure. However, differences in adaptive abilities within the Bufotes and Pelophylax species complexes have not been adequately explored, particularly in light of the recent taxonomic changes.

## 3. Habitat Variations Between Green Toads and Water Frogs in Israel

In Israel, the green toad occupies diverse habitats, ranging from coastal plains and Mediterranean scrublands to arid deserts. The species’ ability to adapt to different environmental conditions has likely contributed to genetic divergence within the indicus in the population (among the individuals within the population) [1,3]. For example, toads in more arid regions may exhibit distinct genetic markers compared to those in wetter, coastal environments. The genetic diversity within the green toad populations in Israel is indicative of historical migration and isolation events [1,8] (Figure 1). The water frog is primarily associated with freshwater habitats, such as rivers, streams, ponds, and marshes. In Israel, this species is found in various aquatic environments, including the coastal plain, the Hula Valley, and other freshwater ecosystems. The phylogenetic variation in water frog populations is influenced by the availability and quality of their aquatic habitats, with populations in isolated or degraded habitats showing signs of genetic divergence. For example, frogs in the northern regions may differ genetically from those in central and southern Israel due to differences in water availability and habitat connectivity [9,10,11,12,13,14,15].

To examine species distribution and the factors influencing it, researchers often utilize the MaxEnt program or leverage previously collected data. For example, Litvinchuk et al. (2024) [16] employed environmental niche modeling to identify glacial refugia and post-glacial colonization pathways for morphologically cryptic marsh frog species (Anura: Ranidae: Pelophylax). Their findings provide insights into the ecological and evolutionary dynamics of these species in response to historical climatic changes.

The study focused on the Upper Oka Basin in Central Russia, examining the factors influencing amphibian presence in local water bodies, with particular attention to the environmental conditions affecting species distribution and spawning habitats. Additionally, research was conducted in the eastern Caucasus, specifically the Caspian region, assessing spawning sites and their significance for the conservation of rare amphibian species in the foothills of the Republic of Dagestan (Askenderov et al., 2018) [17]. Due to the limited number of studies on this topic, it would be beneficial to include a reference link to support further analysis.

## 4. Comparison of Genetic Variation Between Green Toads and Water Frogs in Israel

To analyze the factors that affect the distribution of amphibians, researchers often use methods like multidimensional scaling or review previously published data. For example, a study by Nessi et al. (2023) [18] examined environmental factors influencing amphibian communities in the Southern Apennines. The authors found that various environmental conditions play a crucial role in where different species are located (Nessi et al., 2023) [18].

**Figure 2:**
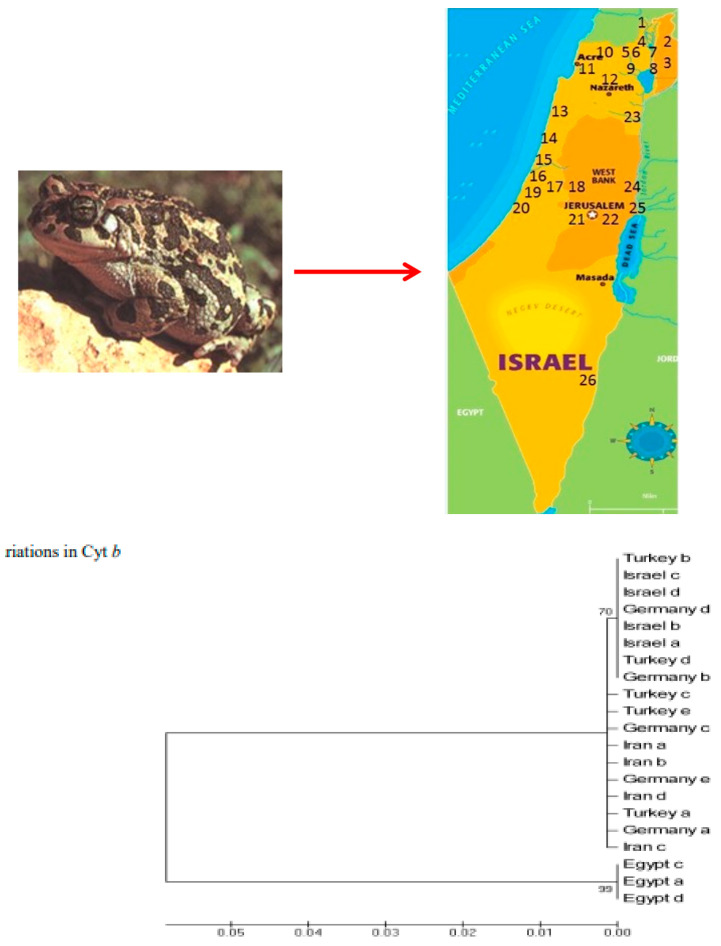
The map show the places in Israel that green toads were found in Israel (1–26). The Cyt b gene was measured for sample 21. Nucleotide similarity and divergence analysis of 21 Cyt b sequences of green toad species from a single location in Israel and 4 other countries: Egypt, Turkey, Germany, and Iran, adapted from [3].

**Figure 3:**
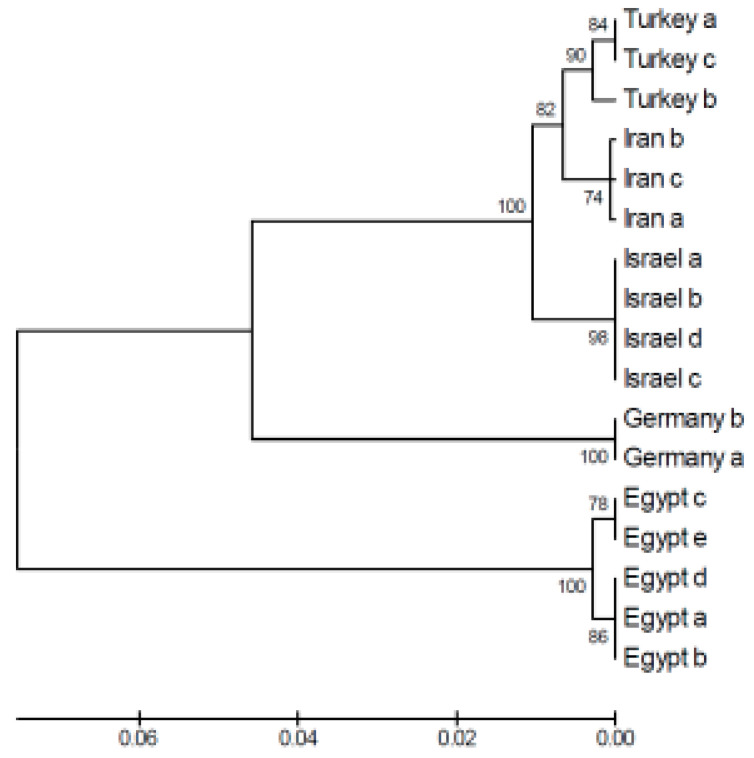
Nucleotide similarity and variation among 17 control region sequences of green toads from sites in Israel and 4 other countries: Egypt, Turkey, Iran, and Germany, adapted from [3].

The green toad exhibits significant genetic variation across its range in Israel, largely due to the species’ wide distribution across diverse habitats. Studies have shown that the green toad in Israel belongs to a species complex with multiple cryptic species, each adapted to different environmental conditions. The genetic differentiation among populations is driven by both geographical isolation and ecological factors, such as the availability of breeding sites and the aridity of the habitat as described previously by [3,19,20,21] (Figure 2). Genetic analyses indicate that populations in the northern and central regions of Israel are more genetically diverse than those in the southern desert regions, likely due to more stable and varied habitats in the north. A comparative analysis of molecular variance (AMOVA) of cytochrome b (Cyt b) and D-loop (Figure 3) fragments from Israeli locations with those from four out-groups showed the highest variance among different regions within Israel. The proportion of total genetic variance among regions in Israel was relatively low and not statistically significant. Random amplified polymorphic DNA (RAPD) cluster analysis for the classification of green toads identified a subgroup consisting of seven populations from the northernmost areas and three populations bordering the southern and eastern deserts of Israel. An amplified fragment length polymorphism (AFLP) analysis grouped all individuals into a single cluster. According to the AMOVA test conducted on Israeli sites using GenAl, the genetic variation in green toads was 2% among regions, 8% among populations, and 90% within populations, as previously described by [3,19,20,21].

A study of the ecological and molecular variations in water frogs from habitats in different locations at different altitudes in northern Israel was conducted. The research involved a series of field and laboratory observations over 4 years, focusing on aquatic habitats to assess the ecological conditions of the breeding sites chosen by the water frog [1]. The duration of larval growth varied among the different populations and breeding sites. In ponds such as Lehavot and Fara, water availability lasted from winter until late summer, when the ponds would dry up. The size of the ponds decreased from 1000 m^3^ to 0 m^3^, while the volume of the spring (Navoraya) remained relatively stable at 3–5 m^3^. Temperatures in these habitats ranged between 5 and 30 °C, with more rapid temperature changes occurring in the ponds than in the spring water. Dissolved oxygen levels varied between 0 and 120% saturation, being higher in winter and lower in summer. The pond water had a higher pH (8–9.5) than the spring water (7.5–8), whereas conductivity remained relatively constant in Navoraya Spring and Fara Pond from January to October. However, in Lehavot Pond, conductivity differed significantly [1].

**Figure 4:**
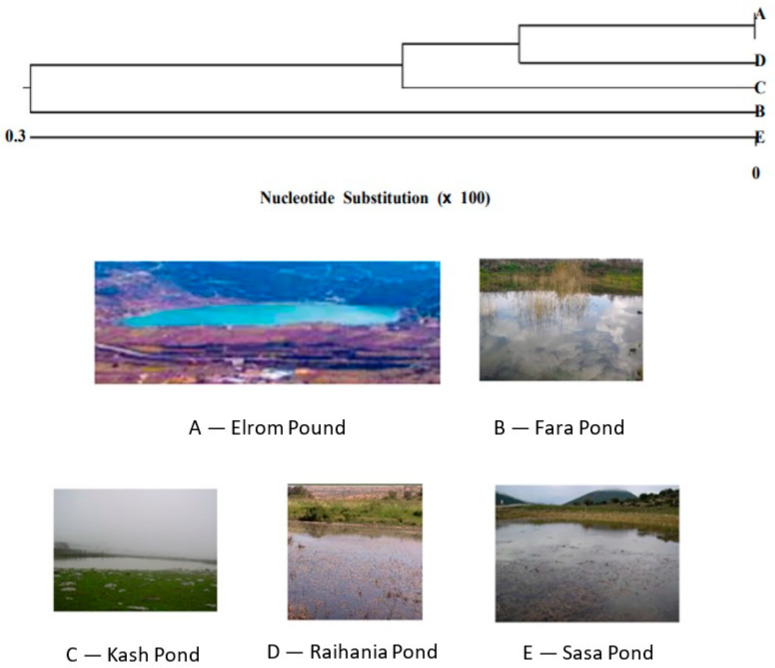
Unrooted phylogenetic tree and percentage identity of the partial Cyt b fragment based on the nucleotide sequence of the Levant water frog. The length of each branch pair illustrates the distance between the sequences, with the units at the bottom representing the number of substitution events. The phylogenetic tree was generated using CLUSTALW in the MegAlign program (DNASTAR). The length of the branches reflects the evolutionary distance. B. Fara Pond. C. Kash Pond. D. Raihania Pond. E. Sasa Pond, adapted from [12,22].

**Figure 5:**
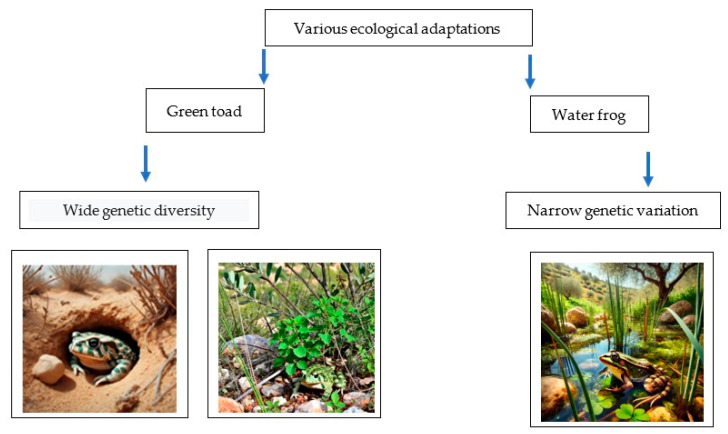
A qualitative model that demonstrates the relationship between genetic variation and adaptation to different environments. Wide genetic variation and distribution confirm adaptation to different environments in Israel (the green toad), and narrower genetic variation (the Levant water frog) reflects narrower area distribution, adapted from [1].

The comparison of genetic diversity between the populations of the green toad and the water frog in Israel can highlight significant differences in adaptation to different environments, genetic histories, and ecological threats (Figure 4 and Figure 5).

Overall, the green toad is widely distributed in Israel, inhabiting both terrestrial and aquatic environments. Genetic diversity in green toad populations is likely influenced by environmental heterogeneity and population fragmentation due to landscape barriers, such as desert areas and mountains. Studies suggest that green toad populations exhibit moderate to high genetic diversity, resulting from varying environmental conditions and adaptation to different habitats [1]. The water frog primarily inhabits freshwater habitats such as streams, ponds, and river deltas. Genetic diversity in water frog populations may be lower compared to the green toad, as it is more dependent on stable water sources. Ecological barriers, such as isolated water bodies, can lead to the development of genetically isolated populations [1]. The proposed model summarizes the key variables between the two amphibian species (5). The first is that the green toad adapted to different terrestrial conditions, from the Mediterranean region to the desert area. The second is that the Levant water frog adapted solely to aquatic life. This summary in the model of the main variables contributes to understanding the factors necessary for amphibians to adapt to terrestrial habitats, and highlights these variables based on various studies from the Mediterranean gradient to the desert region [1].

## 5. Reproduction

The reproductive behavior of the green toad (*Bufo viridis*) in Israel is influenced by various environmental factors, particularly temperature and rainfall. The following gives an overview of their reproductive behavior.

### 5.1. Breeding Season

Breeding in late winter and spring in the cold areas in the north of Israel, and mainly in winter in the south of the country, is related to rain and the appearance of ephemeral water bodies. Green toads in Israel typically breed during the spring, coinciding with the rainy season [1]. The onset of breeding is triggered by rising temperatures and the availability of water bodies created by rainfall. Such winter ponds are, for example, the Sasa pond, which has been described in many studies [9]. In some areas, breeding may continue into the early summer depending on local conditions [1,9,10,23].

### 5.2. Breeding Sites

The toads select shallow, temporary water bodies such as ponds, ditches, or even large puddles as breeding sites. These sites are preferred because they provide a relatively safe environment for eggs and larvae, reducing the risk of predation from aquatic predators [1].

### 5.3. Mating Behavior

**Figure 6:**
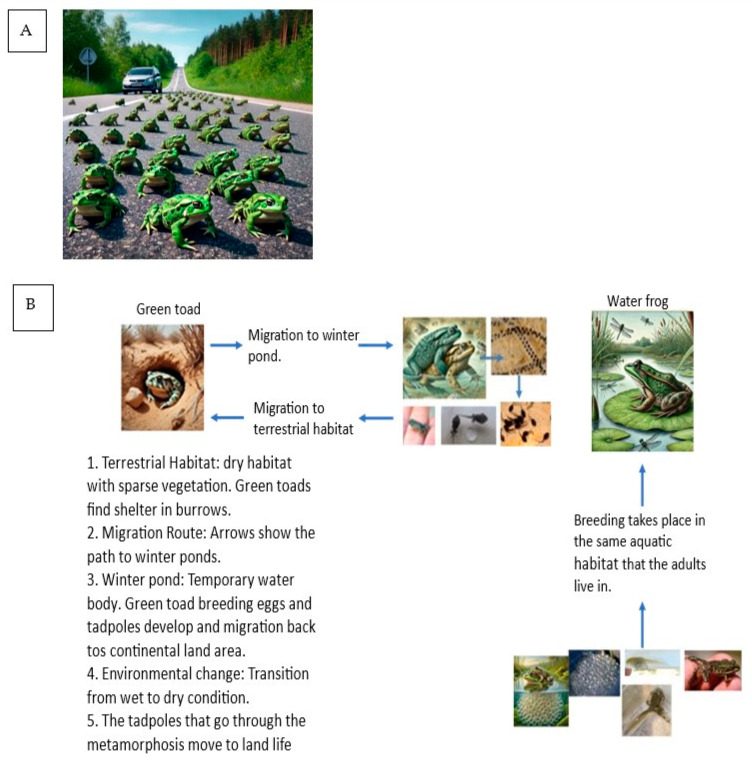
(**A**). Green toads migrate to breeding and spawning areas during the rainy season. During this migration, the toads cross roads, and are at risk of being run over [1]. (**B**). The primary difference between the green toad and the water frog is that the former migrates from terrestrial habitats to water bodies, whereas the latter remains in its aquatic habitat throughout its life cycle, adapted from [1].

Most of the research on the movements of the green toad has been conducted on the toad species in Europe. In Israel, there are mainly observations rather than precise measurements. The green toad lives a solitary life, and migration is primarily to breeding sites. The distances traveled can be several kilometers, and most of the information is based on the distances between the toads and their breeding sites. Both males and females move to water bodies that are used for breeding. The migration period is in winter and spring, so the toads in Israel are not at risk of desiccation during migration. Studies indicate that green toads can travel varying distances to reach breeding sites, 300 m to 1 km on average. In some cases, individuals have been recorded to travel 2 km or more. These long-distance migrations are typically observed in fragmented habitats where breeding sites are scarce. Research conducted in Europe has shown that green toads move between 500 m and 1 km to reach breeding sites. For example, a study in Hungary reported average distances of about 800 m [24]. The distance traveled can also vary seasonally. During the breeding season, toads are known to move more frequently and over longer distances than at other times of the year when they are less mobile and more localized (Figure 6A) [25].

During the breeding season, males congregate at the breeding sites and begin calling to attract females. The call is a distinctive, high-pitched trilling sound. Males compete for the attention of females, and the intensity of their calls often influences mate selection.

Green toads typically lay between 1000 and 5000 eggs in a single clutch. These eggs are often laid in shallow water bodies, where they can develop into tadpoles before eventually metamorphosing into adult toads. The water frog typically lays between 1000 and 3000 eggs in a single clutch. These eggs are usually deposited in shallow water, where they develop into tadpoles before transforming into adult frogs (Figure 6).

## 6. Tadpole Growth and Metamorphosis in Water Frogs and Green Toads

In Israel, the hatching times for the eggs of the green toad and the water frog can be influenced by local environmental conditions, including temperature and water quality. Green toad eggs typically hatch within 7 to 14 days after being laid. The exact timing can vary based on water temperature and other environmental factors. Warmer temperatures usually accelerate the development and hatching process. Water frog eggs generally hatch within 10 to 15 days after being laid. Similarly to the green toad, this period can be influenced by water temperature and other conditions, with warmer temperatures speeding up the process [9,10,26].

**Figure 7:**
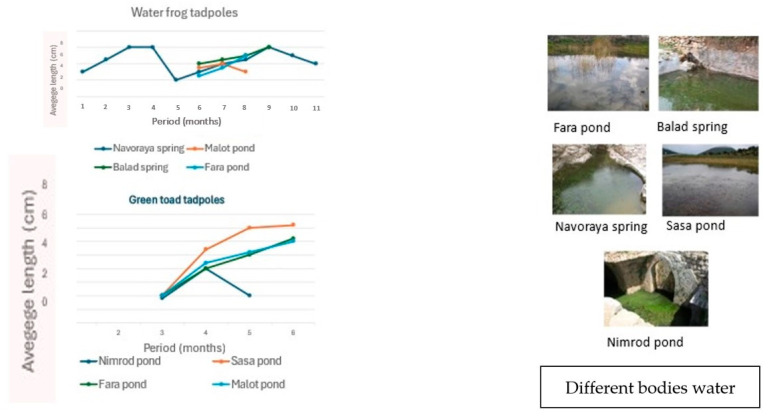
Dimensions of water frog and green toad tadpoles during the growth period in different bodies of water in northern Israel. The findings were collected from several studies summarized in Degani’s 2024 work, adapted from [1].

In both cases, these timeframes are typical for the species, but actual hatching times can vary based on specific local conditions in Israel, such as seasonal temperature fluctuations and habitat characteristics. The periods of tadpole growth and complete metamorphosis are very different (Figure 7).

In Israel, tadpole growth in different bodies of water varies. Green toad tadpoles are primarily found in ponds during the winter and spring, whereas water frog tadpoles are present mainly from spring to summer, and in certain ponds, they can be found year-round (Figure 7). The dimensions of water frog and green toad tadpoles during their growth period also vary across different water bodies in northern Israel. Green toad tadpoles are found in the water for a short period, up to 3 months, whereas water frog tadpoles remain in the water for a longer period, up to 7 or 8 months. Sometimes, these two species coexist in the same habitat, such as a pond that has water for most or all of the year. In winter ponds, where the water remains for 1 to 3 months, only green toad tadpoles can grow and complete their metamorphosis. There are, thus, two prominent differences between green toad tadpoles and water frog tadpoles: green toad tadpoles inhabit water bodies during the winter and metamorphose within 1 to 3 months, which is an adaptation to extreme conditions; water frog tadpoles, on the other hand, have a longer growth period that includes summer, allowing them to survive in stable water bodies for an extended period or for the entire year [9,10,26].

## 7. Different Physiological Adaptations to Terrestrial Life of Green Toads and Water Frogs

### 7.1. Green Toad

The green toad is adapted to arid environments and has developed several physiological mechanisms to cope with drought, as follows:

Green toads (*Bufo viridis*) have specialized skin that allows them to efficiently store water, enabling them to survive long periods of dryness. This adaptation is particularly crucial in environments where water availability fluctuates, such as in hot or arid climates. When faced with dry conditions, these toads can enter a state of torpor or estivation. During this state, their metabolic activity significantly decreases, helping them conserve water. While in estivation, green toads often burrow into the ground or hide beneath vegetation to avoid heat and desiccation, thereby maintaining their body’s moisture. This process of estivation is a remarkable example of adaptation to dry climates, allowing the toads to survive for months without water. When conditions improve and water becomes available again, they can return to full activity. This ability highlights the resilience of organisms and their capacity to thrive even in challenging environments. Their skin secretes a layer of mucus that reduces water loss through evaporation. This adaptation helps them retain moisture during dry periods [27]. Several studies, including [14,28,29], have emphasized the crucial role of urea accumulation in maintaining osmotic balance. Urea acts as a key solute in regulating osmotic pressure in the body and adjusting the overall osmotic balance. During periods of dehydration, when the production of urine is reduced, there is a notable increase in urea concentration in the body’s fluids. This accumulation is partly due to a rise in net urea synthesis, which further contributes to the elevated levels of urea observed during dehydration [14]. Other research has also shown that urea accumulation is an essential mechanism for managing osmotic stress in various amphibians [1].

### 7.2. Water Frog

In contrast, the water frog, which typically inhabits more mesic or aquatic environments, has different strategies for dealing with drought.

Water frogs rely heavily on aquatic habitats and have less capability for water storage in their bodies. They are more vulnerable to changes in water availability, and are likely to seek out water bodies that last through the dry periods [30].

Water frogs exhibit behavioral adaptations, such as migration to wetter areas and burrowing into the mud to escape extreme drought conditions [31].

**Figure 8:**
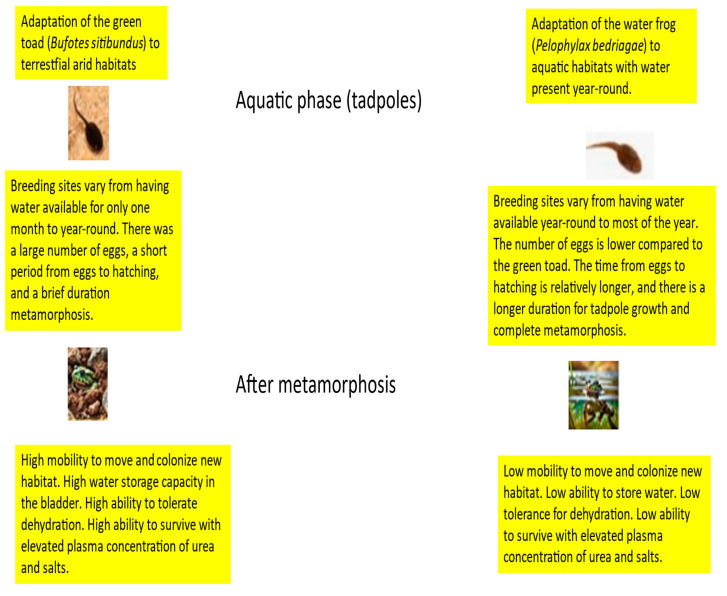
A qualitative model describing the features for adaptation to a semi-arid or arid habitat (the green toad) compared to a semi-aquatic species (the water frog) adapted to a habitat where water is present most or all of the time. The adaptation of tadpoles to winter puddles is evident in their high egg-laying capacity, rapid growth, and swift completion of metamorphosis, as seen in the acute phase model of the green doad. After metamorphosis, the green toad exhibits adaptations for terrestrial life, including water storage and the ability to accumulate urea, which enables a high osmotic pressure in body fluids, facilitating water absorption from the soil.

Overall, while the green toad has evolved physiological adaptations for water conservation and drought resilience, the water frog is more dependent on available water bodies and employs behavioral strategies to cope with drought [1]. A qualitative model describing the adaptation of amphibians to life in semi-arid and arid regions through a comparison of different variables in green toads and water frogs in Israel is presented in Figure 8.

## 8. Conclusions

This article highlights what is known about amphibians regarding habitat adaptation, which must occur in both life stages: the larval stage, primarily in water, focusing on growth rate and metamorphosis, and the adult stage. This topic is particularly interesting in areas transitioning from the Mediterranean to desert regions. In the Mediterranean area, species adapted to dry regions primarily adjust to irregular ecological niches, such as seasonal winter ponds during the larval stage and land areas without water during the terrestrial stage. In contrast, species living in habitats with stable year-round water bodies adapt both before and after metamorphosis. There are still open questions that require further research to explain the differences, e.g., in distribution between the two sexes.

## Data Availability

Raw sequence reads are deposited in the SRA database (BioProject PRJNA1102721). The assembled transcriptome file is available upon request from the author.
